# Miconazole Mitigates Acetic Acid-Induced Experimental Colitis in Rats: Insight into Inflammation, Oxidative Stress and Keap1/Nrf-2 Signaling Crosstalk

**DOI:** 10.3390/biology11020303

**Published:** 2022-02-13

**Authors:** Ifat A. Alsharif, Hany M. Fayed, Rehab F. Abdel-Rahman, Reham M. Abd-Elsalam, Hanan A. Ogaly

**Affiliations:** 1 Biology Department, Jamoum University College, Umm Al-Qura University, Makkah 21955, Saudi Arabia; eesharif@uqu.edu.sa; 2 Pharmacology Department, Medical Research and Clinical Studies Institute, National Research Centre, Dokki, Giza 12622, Egypt; drhany_fayed2000@yahoo.com; 3 Department of Pathology, Faculty of Veterinary Medicine, Cairo University, Giza 12211, Egypt; rehammahmoudpathology@gmail.com; 4 Department of Chemistry, College of Science, King Khalid University, Abha 61421, Saudi Arabia; ohanan@kku.edu.sa; 5 Department of Biochemistry, Faculty of Veterinary Medicine, Cairo University, Giza 12211, Egypt

**Keywords:** miconazole, sulfasalazine, ulcerative colitis, inflammatory bowel disease, oxidative stress

## Abstract

**Simple Summary:**

The protective effect of miconazole, sulfasalazine (as a reference drug) and their combination on acetic acid (AA)-induced ulcerative colitis (UC) in a rat model was investigated. Pretreatment with miconazole significantly reduced wet colon weight and macroscopic scores, accompanied by a significant amelioration of the colonic architecture disorder. Miconazole and/or sulfasalazine revealed protective effects on AA-induced ulcerative colitis via activation of the Nrf2 pathway that improved the antioxidant defense system against oxidative stress and inflammation in UC, as demonstrated by the alleviation of colonic immunopathology, suppression of malondialdehyde (MDA) and elevation in GSH, SOD and HO-1, as well as downregulation of the levels of TNF-α, IL-6 and CRP and upregulation of the IL-10 level. Therefore, miconazole alone—particularly the high dose—or in combination treatment with sulfasalazine was the most efficient candidate compared with sulfasalazine alone and may be an alternative strategy for the treatment of UC.

**Abstract:**

Ulcerative colitis (UC) is the most common type of inflammatory bowel disease, characterized by oxidative stress and elevated pro-inflammatory cytokines. Miconazole is an azole antifungal that stimulates the expression of antioxidant enzymes via Nrf2 activation, which consequently inhibits ROS formation and NF-κB activation. Hence, the present study aimed to investigate the protective effect of miconazole, sulfasalazine (as a reference drug) and their combination on acetic acid (AA)-induced UC in a rat model which was induced by intra-rectal administration of 4% AA. Rats were pretreated with miconazole (20 and 40 mg/kg, orally) or sulfasalazine (100 mg/kg, orally), or their combination (20 mg/kg miconazole and 50 mg/Kg of sulfasalazine, orally). Pretreatment with miconazole significantly reduced wet colon weight and macroscopic scores, accompanied by a significant amelioration of the colonic architecture disorder. Moreover, the treatment also significantly decreased the malondialdehyde (MDA) level and prevented the depletion of superoxide dismutase (SOD) activity and GSH content in inflamed colons. Additionally, the treatment showed suppressive activities on pro-inflammatory cytokines, including tumor necrosis factor-α (TNF-α), interleukin-6 (IL-6) and C-reactive protein (CRP), and upregulated the anti-inflammatory cytokine interleukin-10 (IL-10). Moreover, the treatment upregulated the protein levels of Nrf-2 and heme oxygenase-1 (HO-1) in the colon tissue. Taken together, miconazole is effective in alleviating AA-induced colitis in rats, and the mechanism of its action is associated with the activation of Nrf2-regulated cytoprotective protein expression.

## 1. Introduction

Inflammatory bowel disease (IBD) is one of the most serious health problems worldwide, according to the WHO. Ulcerative colitis (UC) and Crohn’s disease (CD) are the two main types of IBD [1]. IBD has become a worldwide health issue affecting millions of patients [2]. UC is characterized by neutrophil accumulation within the colonic mucosa and generation of micro-abscesses, which cause mucosal inflammation and ulcers [3]. Overproduction of reactive oxygen species (ROS) is caused by chronic inflammation, which has been linked to the development of colon cancer [4]. Oxidative stress plays a prominent role in the pathogenesis of UC via the formation of ROS, infiltration of neutrophils and secretion of pro-inflammatory cytokines [5]. The most common cytokines involved in UC pathogenesis are tumor necrosis factor (TNF)-α, interleukin (IL-1β) and interleukin (IL)-6 [6].

Lipid peroxidation is caused by an imbalance between the oxidative stress and antioxidant systems in IBD, which can impair the antioxidant capacity of the cell, resulting in marked inflamed intestinal mucosa and progression of UC [7,8]. Clinically, ROS and nitrogen species (RNS) were found to be overproduced in colitis patients, resulting in LPO of membranes and disruption of DNA and tissue proteins [9]. Nrf2, nuclear factor-erythroid 2-related factor 2, is a crucial transcription factor that promotes cellular homeostasis via boosting antioxidant gene expression and consequently counteracting ROS generation and the pro-inflammatory response in IBD [10]. UC is associated with the downregulation of the Keap1/Nrf2 signaling pathway and the expression of their target genes [11]. Upon exposure to oxidative stress, The KEAP1-Nrf2 complex sequesters inactive Nrf2 in the cytoplasm, which then translocates to the nucleus and mediates the expression of antioxidant genes by binding to antioxidant response elements (AREs), such as NAD(P)H quinone reductase 1 (NQO1), glutathione-s-transferase (GST) and heme oxygenase (HO-1) [12]. Hence, the pharmacological activation of the Nrf2/ARE pathway may be a potential UC treatment option.

Acetic acid (AA)-induced ulcerative colitis is a widely used animal model that closely mimics the pathogenesis of human colitis. This model is marked by the invasion of inflammatory cells into the intestinal tissue, mucosal and submucosal massive tissue necrosis, edema, vascular dilatation and submucosal ulceration [13]. 

Concerning the targeted population, several studies pointed out the effect of drug-induced colitis, associated with drugs frequently indicated for the treatment of cardiovascular and other related diseases, such as nonsteroidal anti-inflammatory drugs. Additionally, patients who are taking chemotherapy are more susceptible to developing ulcerative colitis [14]. Therefore, further studies are recommended on the usage of miconazole as a preventive strategy against the development of ulcerative colitis in those cases, as well as a combination therapy with sulfasalazine, which is used for the treatment of rheumatoid arthritis, ulcerative colitis and Crohn’s disease and is likely to develop undesirable side effects on the gastrointestinal tract [15]. 

Miconazole, (±)-1-(2-(2,4-dichlorobezyloxy)-2-(2,4-dichlorophenyl)ethyl)-1H-imidazole, is an antimycotic drug that belongs to a family of drugs called the azole family [16]. Miconazole is an antifungal medication that pharmacologically inhibits sterol demethylase and cell wall production by attaching to a fungus-specific binding site against ergosterol biosynthesis [17]. It has been used as an adjuvant therapy to reduce mortality in cancer patients with neutropenia [18]. Further, miconazole shows protective properties against oxidative stress as well as chemo-preventive properties in breast and bladder cancer via activation of the Nrf2 pathway [19]. This evidence indicates that miconazole may be one of the suitable candidates for counteracting ulcerative colitis following acetic acid (AA) injection. Therefore, this study was designed to evaluate the efficacy of miconazole against AA-induced UC in rats and to explore some of the involved mechanisms. 

## 2. Materials and Methods

### 2.1. Animals

Approval of the animal experimental protocol by the Ethical Committee for Medical Research, at the National Research Centre in Egypt, was obtained (approval number: MREC-4419102021). About forty-two adult male Sprague Dawley rats weighing 150–200 g were purchased from the Animal Facility of the National Research Centre, Egypt. Animals were housed in standard cages, under pathogen-free conditions, and maintained under a controlled room temperature and under normal dark–light cycles. Animals were provided with standard food (commercial pellet diet from Al-Marwa for Animals Feed Manufacturing, Cairo, Egypt) and water ad libitum. Rats were allowed to adapt to these conditions for 2 weeks before beginning the experimental protocol. Experiments were performed according to the National Regulations of Animal Welfare and the Institutional Animal Ethical Committee (IAEC).

### 2.2. Drugs and Chemicals

Acetic acid (AA) was obtained from CID Pharmaceutical Co. (Giza, Egypt). Miconazole was obtained from Amriya Pharmaceutical Industries (Alexandria, Egypt), and sulfasalazine was purchased from Minapharm Co. (Cairo, Egypt). All other chemicals were of the highest purity and analytical grade. Enzyme-linked immunosorbent assay (ELISA) kits for the determination of TNF-α, IL-6, IL-10, C-reactive protein (CRP), malondialdehyde (MDA), superoxide dismutase (SOD) and reduced glutathione (GSH) were purchased from R&D Systems, Inc. (Minneapolis, MN, USA).

### 2.3. Induction of Colitis

Colitis was induced by injecting 2 mL of 4% AA into the rectum through a polyurethane tube for enteral feeding (2 mm in diameter) that was inserted to a depth of 4.5 cm. To prevent solution leakage, the rats were held in the Trendelenburg position throughout rectal instillation and for 1 min following instillation [20].

### 2.4. Experimental Design

After the acclimatization period, the rats were classified into six groups (seven rats/group) as follows; The 1st group served as negative control rats that were injected with vehicle (DW), orally. The 2nd group was the untreated ulcerative colitis-induced group in which the rats were injected with acetic acid (AA) intra-rectally (2 mL of 4% (*v*/*v*) in 0.9% NaCl) on day 8. The 3rd and 4th groups were assigned as the ulcerative colitis-induced rats that were orally treated with 20 and 40 mg/kg miconazole, respectively, daily for 7 days and then intra-rectally injected with AA (2 mL of 4% (*v*/*v*) in 0.9% NaCl) on day 8. The 5th group represented the ulcerative colitis-induced rats that were orally treated with sulfasalazine as a reference drug (100 mg/kg/day; orally) for 7 days and then injected with AA intra-rectally (2 mL of 4% (*v*/*v*) in 0.9% NaCl) on day 8. The 6th group represented the ulcerative colitis-induced rats that were orally treated with 20 mg/kg miconazole and sulfasalazine (50 mg/kg/day; orally) for 7 days and then injected with AA intra-rectally (2 mL of 4% (*v*/*v*) in 0.9% NaCl) on day 8.

#### 2.4.1. Collection of Samples

Rats were anesthetized with thiopental sodium (40 mg/kg) 48 h after the intracolonic acetic acid instillation. Colonic segments were taken, cleaned of adipose tissue and rinsed in cold normal saline, and a proper colonic section for macroscopic scoring was implemented. Other portions were used to extract colon homogenates, which were preserved at −80 °C for biochemical investigations, and a third section of colonic segments was stored in 10% neutral buffered formalin for histological and immunohistochemical examinations. 

#### 2.4.2. Colonic Wet Weight Assay

Assessment of the edema degree and the severity of colitis was conducted by recording the weight of the distal 8 cm of the colon of rats [21]. The relative colon weight of each rat was calculated according to El-Alfy et al. [22], using the following formula: “Relative organ weight (g) = [organ weight (g)/body weight (g)] × 100”. Colon specimen wet weight/length (g/cm) ratios were also calculated.

#### 2.4.3. Macroscopic Colonic Damage Scoring

For macroscopic examination of mucosal lesions, colon tissues were divided longitudinally. The macroscopic findings of the colonic mucosa were counted on a scale ranging from 0 to 4 [23]:

(0) There have been no modifications in the macroscopic findings.
(1) Mucosal erythema is the only symptom.
(2) The presence of mild mucosal edema, minor bleeding or minor erosions.
(3) Bleeding ulcers or erosions, as well as moderate edema.
(4) Necrosis, edema and severe ulceration/erosion of the tissue.

### 2.5. Assessment of Colonic Inflammatory Mediators

Pro-inflammatory cytokine (IL-6, TNF-α), anti-inflammatory cytokine (IL-10) and serum C-reactive protein (CRP) levels were assayed in the colonic homogenates using ELISA kits, according to the manufacturer’s instructions.

### 2.6. Assessment of Colonic Oxidative Markers

Malondialdehyde (MDA) content, superoxide dismutase (SOD) activity and reduced glutathione (GSH) concentration were estimated according to the manufacturer’s instructions.

### 2.7. Real-Time Quantitative Polymerase Chain Reaction (RT-qPCR)

Total RNA was extracted from colon tissue samples using TRIzol reagent (Invitrogen; Thermo Fisher Scientific, Inc., Waltham, MA, USA) following the instructions provided [24] and treated with DNase I (Invitrogen). The RNA concentration and purity were estimated using a UV spectrophotometer. Reverse transcription was then performed using SuperScript cDNA Synthesis kit and oligo dT primer (Invitrogen; Thermo Fisher Scientific, Inc.). Real-time PCR was performed with SYBR Green Master Mix (Thermo Fischer Scientific). The qPCR amplification reactions were performed as follows: 10 min at 95 °C, followed by 40 cycles of 10 s at 95 °C and 15 s at 56 °C. Three replicates were applied for each qPCR reaction. The oligonucleotide primers used for qPCR are listed in Table 1. The fold change in the relative mRNA expression of the genes was calculated according to the 2−ΔΔCt method [25] after normalization to β-actin.

### 2.8. Histopathological Examination of Colon Tissue

The harvested colon tissues were fixed in 10% neutral buffered formalin for 24 h, washed with tap water, dehydrated, cleared in xylene, routinely prepared to obtain 5–6 micron-thick paraffin-embedded sections and stained with H&E stain for light microscopy examination [26]. The histopathological scoring system of the colon was performed according to methods described by [27] in the mid-colon area to evaluate inflammatory areas, crypt damage, ulceration and the presence of edema, as described in Table 2, and then the sum of previous lesions was calculated.

For immunohistochemistry, the colon sections of the different groups were deparaffinized in xylene and rehydrated in different grades of alcohol [28,29]. The antigenic retrieval and blocking of the nonspecific background were conducted following the methods of Abu-Elala et al. [30]. The colon sections were washed three times with Tris-buffered saline (TBS) and then incubated with one of the following primary antibodies: rabbit polyclonal anti-INOS antibody (ab15323, Abcam, Cambridge, UK) at 1:100 dilution; mouse monoclonal anti-COX2 antibody (sc-19999, Santa Cruz Biotech, TX, USA) at 1:50; mouse monoclonal anti-caspase-3 antibody (sc-56053, Santa Cruz Biotech, Dallas, TX, USA) at 1:50. The primary antisera were replaced with 1 mg/mL BSA (Sigma, Omaha, NE, USA) for negative control slides. Three washes with TBS were performed to remove excess antibody. The tissue sections were incubated with a biotinylated goat anti rabbit and mouse antibody (Thermo Fisher Scientific, Waltham, MA, USA) for 10 min. Three washes with TBS were performed again to remove excess antibody, followed by incubation with secondary antibody Horseradish Peroxidase complex (Agilent DAKO, Santa Clara, CA, USA). DAB (3,3-diaminobenzidine) was used as a chromogen to visualize the immune-stained cells. Tissue sections were stained with Mayer hematoxylin and mounted. INOS, COX2 and Caspase-3 protein expressions were measured and analyzed in stained areas in 7 random fields per group via image analyzer software (Image J, version 1.46a, NIH, Bethesda, MD, USA) [31].

### 2.9. Statistical Analysis

All results are expressed as means ±SE. All of the tissue analysis was conducted on 7 samples, except for the gene expression analysis, which was conducted on 5 samples. Multiple group comparisons were performed using one-way analysis of variance (ANOVA), followed by the Tukey test at *p* ≤ 0.05, except that for lesion score, which was carried out using the Kruskal–Wallis test followed by the Mann–Whitney test. Statistics and graphical presentations were created using GraphPad prism^®^ software (version 6.00 for Windows, San Diego, CA, USA).

## 3. Results

### 3.1. Miconazole Reduced Colon Weight in AA-Induced Colitis

Acetic acid (AA) raised the relative colon weight as compared with the negative control group and significantly increased the wet weight/length ratio in the UC group, comparable with the negative control rats. Treatment with miconazole (40 mg/kg/day), sulfasalazine and their combination significantly decreased the wet weight/length ratio compared with the UC group (Table 3). Meanwhile, treatment with miconazole at a dose of 20 mg/kg/day showed no significant effect on the wet weight/length ratio as compared to the UC group (Table 3).

### 3.2. Miconazole Attenuated Macroscopic Alterations in AA-Induced Colitis

**Figure 1:**
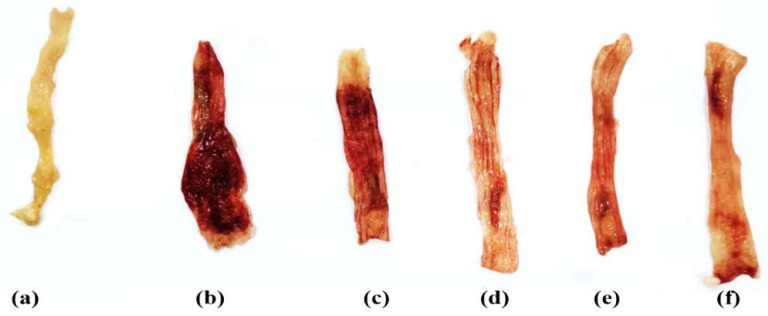
Effect of miconazole, sulfasalazine and their combination on the macroscopic appearance of the colonic mucosa: (**a**) NC, negative control group; (**b**) UC, positive control group; (**c**) MIC-20, miconazole 20 mg/kg/day-treated group; (**d**) MIC-40, miconazole 40 mg/kg/day-treated group; (**e**) SSZ-100, sulfasalazine 100 mg/kg/day-treated group; (**f**) MIC + SSZ, combination therapy-treated group.

In comparison to the negative control rats, acetic acid caused severe edematous inflammation in the colon, with a much higher macroscopic score of colonic damage. The mucosa was ulcerated, edematous and hemorrhagic in appearance (Figure 1, Table 3). Meanwhile, the miconazole (20 and 40 mg/kg/day), sulfasalazine (100 mg/kg/day) and combination therapy-treated groups markedly alleviated the severity of the gross lesion score as compared to the UC group (Figure 1). Additionally, the highest protective effect was shown in the combination-treated group. Macroscopically, the least affected groups were those treated with miconazole 40 mg/kg/day and sulfasalazine 100 mg/kg/day, with no notable difference between them (Figure 1).

### 3.3. Miconazole Alleviated Oxidative Stress in AA-Induced Colitis

The malondialdehyde (MDA) content was assayed as a lipid peroxidation marker in the colons of all rats. The level of colon MDA was markedly elevated in the UC group compared to the control group (Table 4). However, pretreatment of rats with miconazole, sulfasalazine and the combination therapy showed a marked decline in the MDA content in colon homogenates of rats, compared to the UC group. The reduced glutathione (GSH) content and superoxide dismutase (SOD) activity were markedly depleted in the UC group as compared with the negative control rats (Table 4). Interestingly, the colonic tissues of the pretreated groups significantly restored the GSH content and SOD activity compared to the UC group. Improvements in these parameters were remarkable in the combination group amongst the pretreated groups.

### 3.4. Miconazole Downregulated the Pro-Inflammatory and Upregulated the Anti-Inflammatory Mediators in AA-Induced Colitis

The results show that acetic acid injection led to a significant reduction in the levels of IL-10, an anti-inflammatory cytokine, and caused a marked elevation in the pro-inflammatory cytokines TNF-α and IL-6 in rat colons as compared to the negative control group (IL-10; TNF-α; IL-6; Table 5). Meanwhile, pretreatment with miconazole (40 mg/kg), sulfasalazine and their combination significantly elevated the level of the anti-inflammatory cytokine IL-10 and diminished the production of the pro-inflammatory cytokines compared to the UC group (TNF-α; IL-6), as shown in Table 5. Additionally, there was an insignificant relationship between these parameters in the combination group compared to the control group. Likewise, the TNF-α level markedly declined in the sulfasalazine-treated group compared to the UC control, with an insignificant difference compared to the NC control. An improvement in these inflammatory mediators was significantly exhibited in the combination group amongst the other pretreated groups.

### 3.5. Miconazole Upregulated Nrf2 and Heme Oxygenase-1 (HO-1) and Downregulated CRP in AA-Induced Colitis

**Figure 2:**
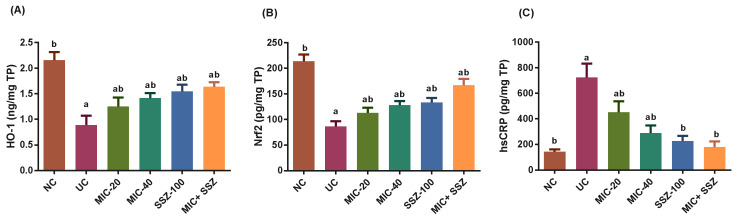
Effect of miconazole, sulfasalazine and their combination on Nrf2 (**A**), HO-1 (**B**) and CRP (**C**) in AA-induced UC. Each group’s values are expressed as the mean ± SEM of seven animals. ^a^ Significantly different at *p* ≤ 0.05 from the negative control (NC) values. ^b^ Significantly different at *p* ≤ 0.05 from the ulcerative colitis control (UC) values.

In the UC group, HO-1 and Nrf2 were significantly downregulated, and CRP was markedly elevated, as compared to the NC control group (Figure 2A–C). Both treatments, miconazole and sulfasalazine, as well as their combination, significantly improved HO-1 and Nrf2 levels and significantly decreased CRP levels compared to the UC group. In comparison to the negative control group, there was no significant difference in CRP levels in the sulfasalazine and combination groups.

### 3.6. Miconazole Amended Histopathological Alterations in AA-Induced Colitis

**Figure 3:**
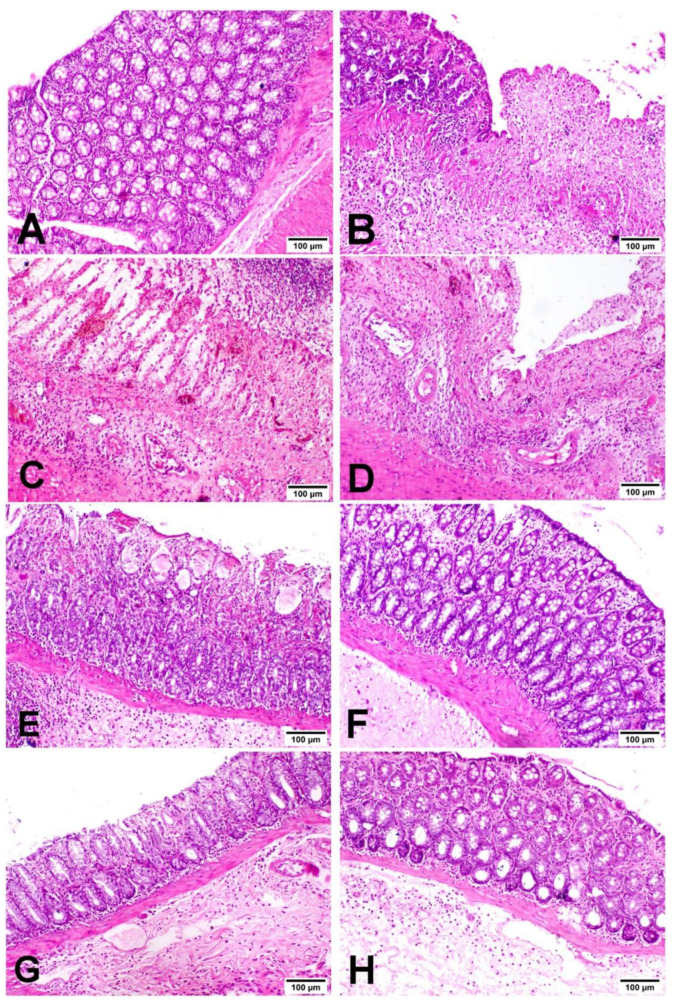
Photomicrograph of colons of different groups stained with H&E stain. (**A**) NC group showing a normal histological finding of the colon. (**B**) UC group showing area of ulcerative colitis with complete destruction of the colon mucosa. (**C**) UC group showing complete crypt necrosis, inflammatory cell infiltration in the mucosa and submucosa and congestion of blood vessels. (**D**) UC group showing complete necrosis of the mucosa with submucosal fibrous connective tissue proliferation with mononuclear inflammatory cell aggregation. (**E**) MIC-20 showing necrosis of some crypts with a moderate inflammatory reaction in the mucosa and submucosa. (**F**) MIC-40, (**G**) SSZ-100 and (**H**) MIC + SSZ groups showing minimal inflammatory reaction in the mucosa and submucosal edema.

**Figure 4:**
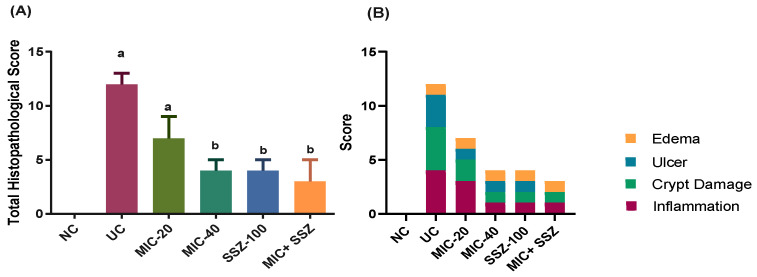
Histopathological lesion scoring of AA-induced ulcerative colitis. (**A**) Total scores, and (**B**) scores including the evaluated parameters (inflammation, crypt damage, ulcer and edema); *n* = 7, median with interquartile ranges, ^a^ indicates a significant difference compared to the NC group at *p* ≤ 0.05, and ^b^ indicates a significant difference compared to the UC group at *p* ≤ 0.05.

The histopathological examination of colons in the different experimental groups revealed that colons of the NC group exhibited a normal histological architecture of the mucosa, submucosa and muscularis (Figure 3A). The colonic crypts were lined by absorptive columnar epithelium with numerous goblet cells, and lamina propria contained few mononuclear inflammatory cells. On one hand, the UC group exhibited severe histopathological changes in the form of a typical picture of ulcerative colitis (Figure 3B–D). There were multiple areas of erosion and ulceration with complete necrosis of the mucosa, submucosa and muscularis of the bowel wall. Ulcers were at different stages of healing; some ulcers were completely replaced by granulation tissue, with pyogranulome formation in the submucosa. The remaining colonic mucosa and submucosa showed significant crypt distortion with apoptosis and necrosis of the lining epithelium with marked loss of goblet cells, and the lamina propria and submucosa were massively infiltrated with neutrophils and mononuclear cells with obvious submucosal edema and hemorrhage. On the other hand, the groups treated with MIC-40, SSZ-100 and MIC + SSZ showed significantly reduced ulcer formation, mucosal injury and colonic inflammation induced by AA, while MIC-20 had no significant effect, as shown in Figure 3E–H. The MIC-20 group showed necrosis of some crypts with a moderate inflammatory reaction in the mucosa and submucosa (Figure 3E). However, the MIC-40- and MIC + SSZ-treated groups exhibited minimal inflammatory reaction in the mucosa and submucosa without any feature of tissue destruction (Figure 3F,H). In addition to that, crypts had a normal architecture with intact vacuolated goblet cells and less apoptotic or necrosed columnar epithelium. Concerning the histopathological lesion scoring of ulcerative colitis, the UC group showed a significant elevation in all parameters as well as the total score compared to the NC group, and the MIC-40, SSZ-100 and MIC + SSZ groups exhibited a significant reduction in such parameters compared to the UC group (Figure 4). Treatment with MIC-20 did not significantly affect any of the investigated parameters as compared with the UC group (Figure 4).

### 3.7. Miconazole Downregulates INOS, COX2 and Caspase-3 Protein Expression in AA-Induced Colitis

**Figure 5:**
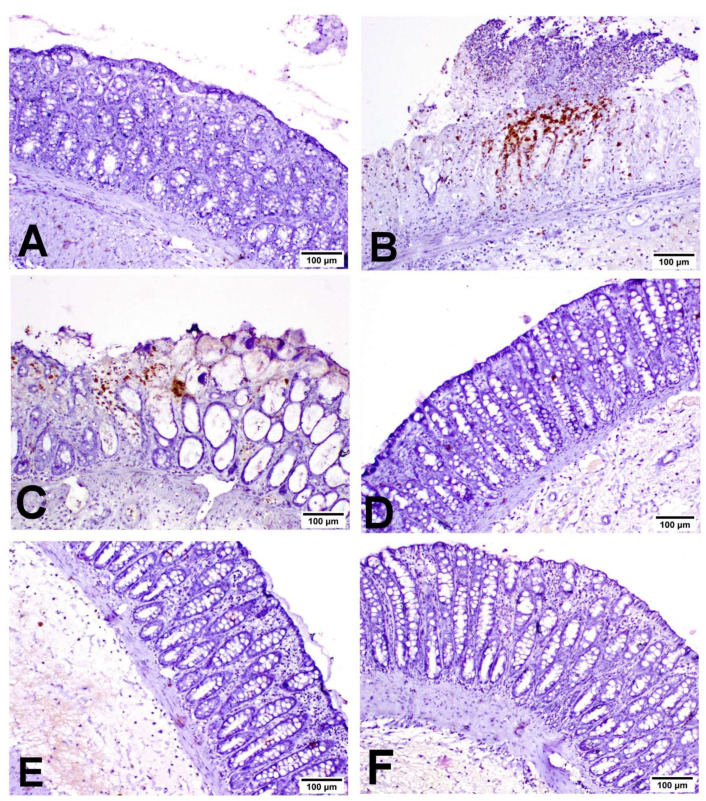
Immunohistochemistry of INOS expression in the colon of the different groups: (**A**) NC group; (**B**) UC group; (**C**) MIC-20 group; (**D**) MIC-40 group; (**E**) SSZ-100 group; (**F**) MIC + SSZ group.

**Figure 6:**
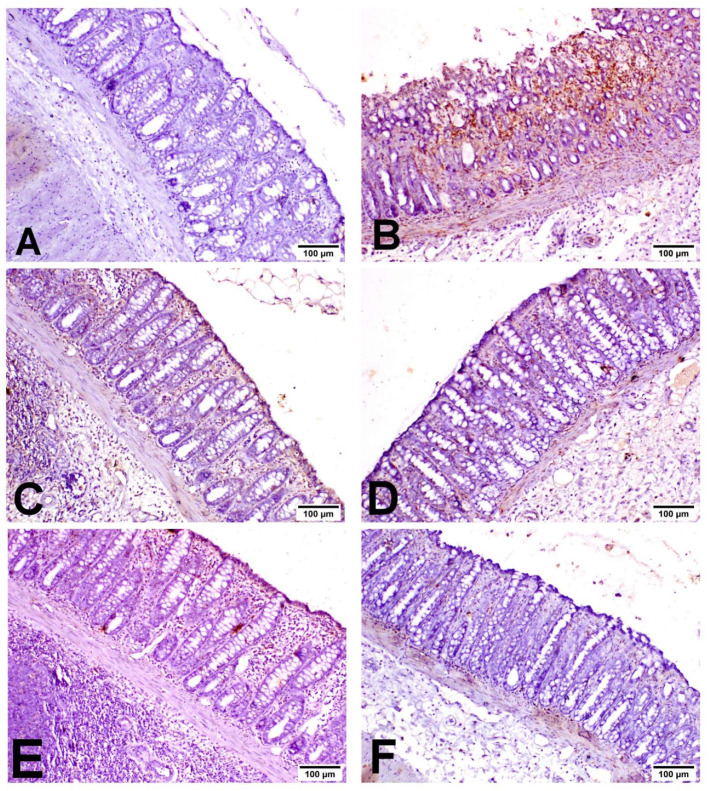
Immunohistochemistry of COX2 expression in the colon of the different groups: (**A**) NC group; (**B**) UC group; (**C**) MIC-20 group; (**D**) MIC-40 group; (**E**) SSZ-100 group; (**F**) MIC + SSZ group.

**Figure 7:**
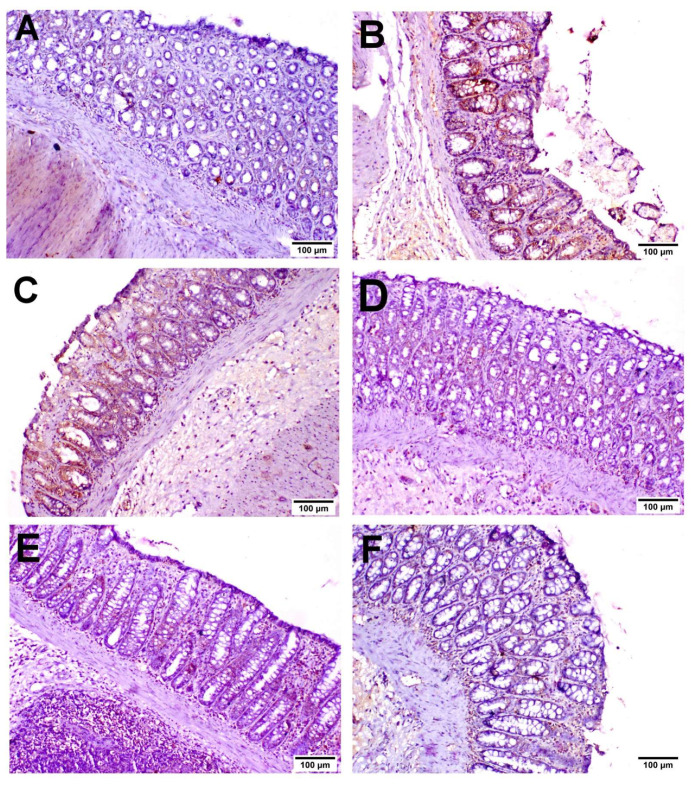
Immunohistochemistry of Caspase-3 expression in the colon of the different groups: (**A**) NC group; (**B**) UC group; (**C**) MIC-20 group; (**D**) MIC-40 group; (**E**) SSZ-100 group; (**F**) MIC + SSZ group.

**Figure 8:**
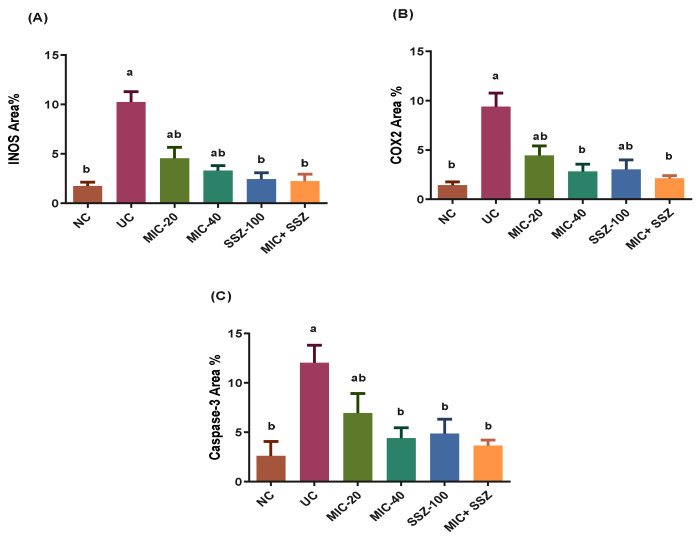
Effect of miconazole, sulfasalazine and their combination on INOS, COX2 and Caspase-3 in AA-induced UC. (**A**) INOS positively stained area percentage. (**B**) COX2 positively stained area percentage. (**C**) Caspase-3 positively stained area percentage. Each group’s values are expressed as the mean ± SEM of seven animals. ^a^ Significantly different at *p* ≤ 0.05 from the negative control (NC) values. ^b^ Significantly different at *p* ≤ 0.05 from the ulcerative colitis control (UC) values.

As shown in Figure 5, INOS was expressed in inflammatory cells with little expression in the lining epithelium. COX2 was expressed mainly in the epithelial lining and mononuclear inflammatory cells in the lamina propria (Figure 6). Caspase-3 was expressed in the lining epithelium and internal crypts, and it was evaluated in non-ulcerative areas (Figure 7). A significant elevation in INOS, COX2 and Caspase-3 protein expressions was recorded in the UC-treated group compared to the NC group, and a significant reduction in these expressions was observed in the MIC-20, MIC-40, SSZ-100 and MIC + SSZ groups when compared with the UC group (Figure 8).

### 3.8. Miconazole Activates Nrf2/HO-1 Signaling in AA-Induced Colitis

**Figure 9:**
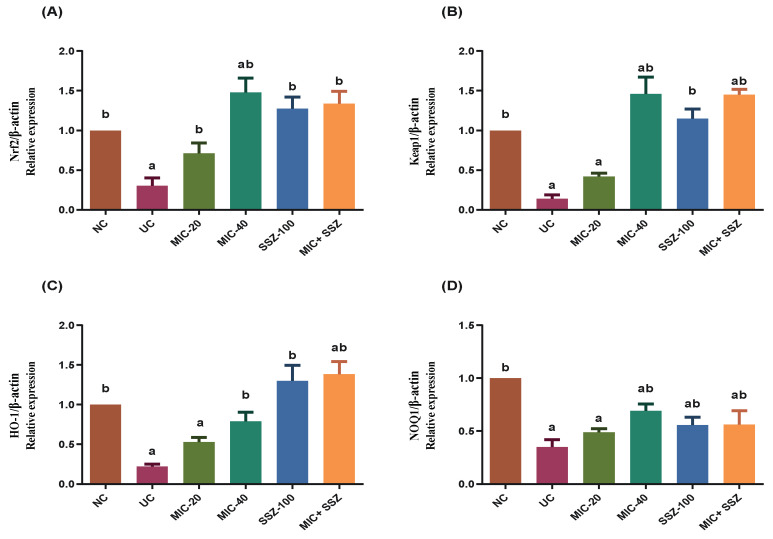
Effect of miconazole, sulfasalazine and their combination on mRNA expression of Nrf2 (**A**), Keap-1 (**B**), HO-1 (**C**) and NOQ1 (**D**) in AA-induced UC. Data are expressed as fold changes of negative control value ± SEM (*n* = 5) after normalization by β-actin. ^a^ Significantly different at *p* ≤ 0.05 from the negative control (NC) values. ^b^ Significantly different at *p* ≤ 0.05 from the ulcerative colitis control (UC) values.

Given that activation of Nrf2/HO-1 signaling could alleviate the allergic responses associated with UC [32,33], we detected the expression of Nrf2 pathway-related genes in the colon tissue by RT-qPCR. According to the results (Figure 9), colon Nrf2 mRNA expression significantly deteriorated in the UC model group to about 31% in comparison to the normal control group. Pretreatment with MIC, SSZ-100 or their combination significantly reversed the AA-induced reduction in colon Nrf-2 expression. Interestingly, in the MIC-20-, MIC-40-, SSZ-100- and MIC + SSZ-treated groups, Nrf2 expression significantly escalated to 91%, 148%, 128% and 134%, respectively, of its normal expression level (Figure 9).

Similarly, colon HO-1, Keap-1 and NOQ1 expressions significantly declined in the UC group to approximately (0.22), (0.14) and (0.35) of their normal expression, respectively (Figure 9). On the other hand, the obtained findings reveal that pretreatment with MIC, SSZ-100 or their combination effectively upregulated the mRNA expression of Nrf2-dependent genes in UC rats, where the expression level of HO-1 was increased by 53%, 79%, 130% and 139% of the normal expression in the groups subjected to MIC-20, MIC-40, SSZ-100 and MIC + SSZ treatments, respectively (Figure 9). The MIC-20, MIC-40, SSZ-100 and MIC + SSZ groups showed increased expression levels of Keap-1 by 43%, 146%, 115% and 145%, and NOQ1 by 49%, 69%, 58% and 56%, of the normal expression levels, respectively (Figure 9).

## 4. Discussion

Ulcerative colitis (UC) is categorized as one of the major forms of IBD, implicating immune, genetic and environmental factors in the initiation and development of colitis [34]. Currently available agents for IBD including corticosteroids, 5-aminosalicylates and immunosuppressants are not entirely effective and also display several adverse effects which limit their long-term use [35]. Hence, there is a clear need for new therapeutic agents that have more efficacy with fewer adverse effects. The present study revealed, for the first time, the protective role of miconazole against an experimentally induced UC model via activating the Keap1/Nrf2 pathway.

Indeed, miconazole is a broad-spectrum azole antifungal against candidiasis, with some activity against Gram-positive bacteria as well [17]. There are few reports of antifungal drugs affecting intestinal mucosal morphology, gut microbiota and immunity [36]. Therefore, further studies are needed to evaluate the influence of the antifungal miconazole on the gut microbiota and immune system.

Several studies showed that the UC rat model via AA intra-rectal injection can closely mimic the human UC pathogenesis and histological features [37,38]. In this research, the rat colon weight was markedly elevated following the intra-rectal injection of AA, and this increase was accompanied by severe macroscopic lesions and ulceration of the colon tissue. Additionally, AA produced severe inflamed edematous colons accompanied by a significant macroscopic scoring of colon lesions as compared with the negative control. As confirmed by histopathological examination, deterioration, ulceration, erosion, congestion, inflammatory cell infiltration, necrosis and goblet cell hyperplasia of the entire colonic mucosa were exhibited after AA administration.

The findings from the current study are in harmony with those revealed in prior studies using the AA-induced UC rat model [39,40]. Pretreatment with miconazole at two different dose levels (20 or 40 mg/kg/day) for 7 days was shown to restore the histopathological deterioration of colon tissue, compared to colitis rats, and markedly alleviated the severity of the gross lesion score as compared to the UC group (Figure 1).

One of the key processes implicated in the pathophysiology of IBD is oxidative stress, which is one of the immune-regulatory variables [41]. It has been recognized that overproduction of both ROS and RNS as well as an imbalance between the generation and elimination of these free radicals via the antioxidant system is associated with the initiation and progression of UC [5]. Oxidative stress resulted in decreased levels of enzymatic superoxide dismutase (SOD) and non-enzymatic glutathione (GSH) antioxidants, which cause lipid peroxidation [42]. GSH is regarded as a free radical scavenger or cellular oxidation inhibitor, and depletion of the GSH content is a marker of oxidative stress [43]. Accordingly, the GSH drop directly resulted in elevation of the MDA level, an end product of lipid peroxidation, which led to polyunsaturated lipid degradation [44]. SOD scavenges the free radicals and controls oxidative stress in different pathways [45]. SOD has the ability to convert superoxide radicals into hydrogen peroxides (H_2_O_2_) and molecular oxygen, while catalase and peroxidases have the ability to convert H_2_O_2_ into water [46]. In the current study, increased oxidative stress was observed in the colitis model, which was confirmed by the elevated MDA level as well as the reduced GSH content and SOD activity in colon tissues. These results are consistent with previous findings [9,47]. In the present study, pretreatment of AA-induced colitis rats with miconazole, particularly the high dose, or its combination with sulfasalazine was able to protect mucosal damage via reducing the MDA levels and restoring the GSH content and SOD activities in the colon tissues. In line with the findings of the current work, the antioxidant efficacy of miconazole has been shown in a previous study by [19], who revealed that the protective findings of miconazole may be attributed to Nrf2 activation by stimulating the expression of antioxidant genes and inhibiting free radical generation.

Furthermore, miconazole significantly inhibited oxidative stress and inflammatory responses in the colon following AA administration in a dose-dependent manner. The effect of pretreatment with miconazole (40 mg/kg/day) was similar to that of the sulfasalazine-treated group. Interestingly, the combined therapy of miconazole and sulfasalazine produced more remarkable protective effects on UC than the use of either agent alone.

The significant role of inflammatory cytokines in the pathogenesis of UC has been reported in numerous studies. An imbalance between the secretion of pro-inflammatory (TNF-α, IL-6, IL-12, IL-23, etc.) and anti-inflammatory cytokines stimulates colon tissue damage and, subsequently, progression of UC disease. Upon immunological dysregulation, tumor necrosis factor-α (TNF-α), which is known as an inflammatory mediator, produced and stimulated apoptosis which, in turn, led to the secretion of IL-1 and IL-6 [48]. In UC patients, there is strong evidence that the increased serum levels of IL-6 have a role in triggering and sustaining the intestinal inflammatory response [49]. Pro-inflammatory cytokines (TNF-α, IL-1, IL-6, IL-9, IL-13 and IL-33) play an essential role in the upregulation of UC progression, while anti-inflammatory cytokines (transforming growth factor-β, IL-10 and IL-37) play a marked role in its downregulation. IL-10 is a pleiotropic immunoregulatory cytokine that plays a key role in the anti-inflammatory effect associated with tissue recovery [50]. The study of these UC immuno-inflammatory pathways might result in the development of a specific novel therapy that could be more effective [51]. Data from the current study reveal that the levels of inflammatory markers, including IL-6, TNF-α and serum C-reactive protein, were significantly increased in colon tissues of experimental UC rats. Additionally, the level of IL-10 was significantly decreased after induction of UC compared to the negative control rats. These findings suggest that the UC group colons were inflamed, which was confirmed by the histopathological results. Further, treatment with miconazole significantly diminished the elevated levels of inflammatory markers, including IL-6, TNF-α and serum C-reactive protein, and enhanced the production of the anti-inflammatory cytokine IL-10 in colon tissues of UC rats. These findings are in accordance with previous studies by [52], who confirmed that miconazole possesses anti-inflammatory activity. Parallel with the study results, [53] found that miconazole treatment could decrease the degree of pro-inflammatory cytokine production and increase the levels of the IL-10 cytokine in a mouse model of sciatic nerve crush injury [53]. Furthermore, miconazole suppressed neuroinflammation in a mouse model of LPS-induced memory loss by inhibiting the activation of NF-κB, a transcription factor that stimulates the secretion of inflammatory cytokines, such as TNF-α, IL-1β and IL-6, in the brain [54].

Notably, the major regulator of the endogenous antioxidant system is Nrf2, which protects cells from oxidative stress agents [55]. Under normal conditions, Nrf2 binds with Kelch-like ECH-associated protein 1 (keap1) which inhibits Nrf2 translocation to the nucleus [56]. Therefore, excessive formation of free radicals dissociates the keap1-Nrf2 complex and a free Nrf2 is translocated to the nucleus, where it binds with small Maf (sMaf) proteins [57]. The heterodimer binds with antioxidant response element (ARE) target genes, promoting the expression of several antioxidant-related genes, including GSH, SOD and HO-1 [56]. Based on this, Nrf2 is a key mediator of the antioxidant defense system as well as an important target for the treatment of UC [58,59]. Heme oxygenase-1 (HO-1) is expressed in the epithelial cells to impair the pro-inflammatory factors and oxidative stress in intestinal disease models [60]. In this study, the Nrf2/HO-1 pathway was significantly downregulated in the UC group and upregulated in the pretreated groups, which indicated that the Nrf2/HO-1 signaling pathway participates in the alleviation of UC pathogenesis. These findings are consistent with the previous findings of [19], who revealed that miconazole could promote Nrf2 activation through the p62-KEAP1 noncanonical pathway as well as via inhibition of Keap1 protein expression. The treatment with miconazole, particularly the high dose, or the combination therapy of miconazole with sulfasalazine was more effective in the upregulation of the Nrf2/HO-1 pathway.

To sum up, the mitigation of the inflammatory responses in the miconazole-treated groups may be attributed to the activation of the Nrf2 pathway. This hypothesis was confirmed in previous studies [11,61], which showed that the activation of Nrf2 could attenuate inflammatory signaling via inhibiting NF-κB translocation into the nucleus. Numerous studies have confirmed the critical role of the transcription factor NF-κB in both the immune and the inflammatory responses. Thus, the activated NF-κB transcription factors stimulate the gene expression of numerous cytokines including IL-6 and TNF-α [62,63]. Therefore, we confirmed that miconazole, particularly at a high dose, and its combination with sulfasalazine alleviated oxidative stress and inflammation in experimental UC through activation of the Nrf2 pathway.

Overall, the current study conclusively proves the potential protection exerted by MIC against AA-induced UC in rats via its antioxidant and anti-inflammatory effects. However, the limitations of our study include the use of only two doses of MIC, therefore missing the minimum number of doses that could be useful for clinical study purposes and for dose–response calculation. Second, this investigation did not adopt a time course of MIC administration. In addition, how MIC regulates the expression of Nrf2 pathway-related proteins also needs further study.

## 5. Conclusions

To conclude, pretreatment with miconazole and/or sulfasalazine seems to have protective effects on AA-induced ulcerative colitis via activation of the Nrf2 pathway that improved the antioxidant defense system against oxidative stress and inflammation in UC, as demonstrated by the alleviation of colonic immunopathology, suppression of MDA and elevation in GSH, SOD and HO-1, as well as downregulation of the levels of TNF-α, IL-6 and CRP and upregulation of the IL-10 level. Therefore, miconazole alone—particularly the high dose—or in combination treatment with sulfasalazine was the most efficient candidate compared with sulfasalazine alone and may be an alternative strategy for the treatment of UC.

## Figures and Tables

**Table 1:** Primers used for RT-qPCR.

Gene	Accession No.	Product Size	Sequence (5′–3′)	
Nrf2	XM_006234398.3	121	F	CACATCCAGACAGACACCAGT
			R	CTACAAATGGGAATGTCTCTGC
NQO1	NM_017000.3	162	F	CTGTGAGGGACTCTGGTCTTTG
			R	CTGAAAGCAAGCCAGGCAAAC
Keap-1	NM_017000.3	190	F	AACTCGGCAGAATGTTACTACCC
			R	CTACGAAAGTCCAGGTCTCTGTCTC
HO-1	NM_012580.2	107	F	ACAGGGTGACAGAAGAGGCTAA
			R	TCAAGAGGAGCAGAAAAAGAACAAG
β-actin	NM_031144.3	97 bp	F	GGTGGGTATGGGTCAG
			R	ATGCCGTGTTCAATGG

Nrf2, nuclear factor erythroid 2-related factor 2; NQO1, NAD(P)H dehydrogenase [quinone] 1; Keap-1, kelch-like epichlorohydrin-associated protein 1; HO-1, heme oxygenase-1.

**Table 2:** Histopathological lesion score system.

**Inflammatory Cell Infiltration**	
0. None	
1. Occasional, limited to submucosa	
2. Significant focal areas in submucosa	
3. Significant focal areas in submucosa and lamina propria	
4. Diffuse large areas in submucosa, around blood vessels and lamina propria	
5. Transmural infiltration from mucosa to muscularis	
**Crypt Damage**	
0. No change	
1. Some crypt damage, spaces between crypts	
2. Goblet cell loss, some shortening of crypts with larger spaces between them	
3. Large areas without crypts	
4. No crypt	
**Ulceration**	
0. None	
1. Small focal ulcers	
2. Frequent small ulcers	
3. Large areas without surface epithelium	
**Edema**	
0. Absent	
1. Present	
Total lesion score	0–13

**Table 3:** Effect of miconazole, sulfasalazine and their combination on relative colon weight, W/L ratio and lesion score in AA-induced UC.

Group	Relative Colon Weight (g)	W/L Ratio (g/cm)	Lesion Score
NC	0.34 ^b^ ± 0.04	0.071 ^b^ ± 0.008	0 ± 0
UC	0.65 ^a^ ± 0.06	0.133 ^a^ ± 0.01	3.52 ± 0.19
MIC-20	0.62 ^a^ ± 0.03	0.129 ^a^ ± 0.007	2.57 ± 0.20
MIC-40	0.53 ^a^ ± 0.01	0.102 ^ab^ ± 0.004	1.71 ± 0.29
SSZ-100	0.53 ^a^ ± 0.02	0.102 ^ab^ ± 0.005	1.86 ± 0.26
MIC + SSZ	0.53 ^a^ ± 0.02	0.106 ^ab^ ± 0.002	1.43 ± 0.20

Each group’s values are expressed as the mean ± SEM of seven animals. ^a^ Significantly different at *p* ≤ 0.05 from the negative control (NC) values. ^b^ Significantly different at *p* ≤ 0.05 from the ulcerative colitis control (UC) values.

**Table 4:** Effect of miconazole, sulfasalazine and their combination on oxidative stress markers MDA (A), GSH (B) and SOD (C) in AA-induced UC.

Group	MDA (nMol/mL)	GSH (nMol/dL)	SOD (U/mL)
NC	8.5 ^b^ ± 0.31	0.9 ^b^ ± 0.03	17.6 ^b^ ± 0.37
UC	13.8 ^a^ ± 0.43	0.6 ^a^ ± 0.02	11.7 ^a^ ± 0.23
MIC-20	11.7 ^ab^ ± 0.4	0.7 ^ab^ ± 0.02	13.0 ^ab^ ± 0.23
MIC-40	11.4 ^ab^ ± 0.23	0.7 ^ab^ ± 0.02	13.9 ^ab^ ± 0.38
SSZ-100	10.7 ^ab^ ± 0.26	0.8 ^ab^ ± 0.01	15.0 ^ab^ ± 0.18
MIC + SSZ	10.0 ^ab^ ± 0.23	0.8 ^ab^ ± 0.01	16.4 ^b^ ± 0.39

Each group’s values are expressed as the mean ± SEM of seven animals. ^a^ Significantly different at *p* ≤ 0.05 from the negative control (NC) values. ^b^ Significantly different at *p* ≤ 0.05 from the ulcerative colitis control (UC) values.

**Table 5:** Effect of miconazole, sulfasalazine and their combination on the anti-inflammatory cytokine IL-10 and pro-inflammatory cytokines IL-6 and TNF-α in AA-induced UC.

Group	IL-10 (pg/mg TP)	IL-6 (pg/mg TP)	TNF-α (pg/mg TP)
NC	262.7 ^b^ ± 16.34	154.6 ^b^ ± 5.84	198.1 ^b^ ± 3.87
UC	145.2 ^a^ ± 3.74	309.6 ^a^ ± 13.75	342.7 ^a^ ± 17.07
MIC-20	176.9 ^a^ ± 6.52	261.0 ^ab^ ± 6.76	286.5 ^ab^ ± 15.63
MIC-40	193.7 ^ab^ ± 8.14	249.2 ^ab^ ± 14.27	254.4 ^ab^ ± 12.96
SSZ-100	224.6 ^ab^ ± 6.65	208.4 ^ab^ ± 13.08	241.8 ^b^ ± 6.16
MIC + SSZ	238.8 ^b^ ± 2.46	166.2 ^b^ ± 3.31	207.6 ^b^ ± 7.88

Each group’s values are expressed as the mean ± SEM of seven animals. ^a^ Significantly different at *p* ≤ 0.05 from the negative control (NC) values. ^b^ Significantly different at *p* ≤ 0.05 from the ulcerative colitis control (UC) values.

## Data Availability

The data presented in this study are available in the open access manuscript.
